# SMARCB1-deficient sinonasal carcinoma: a case report and literature review

**DOI:** 10.1093/jscr/rjab161

**Published:** 2021-04-30

**Authors:** Naoki Yanagawa, Masamichi Suzuki, Ryo Sugimoto, Mitsumasa Osakabe, Noriyuki Uesugi, Kiyoto Shiga, Tamotsu Sugai

**Affiliations:** Departments of Molecular Diagnostic Pathology, Yahaba-cho, Shiwa-gun, Iwate, Japan; Departments of Molecular Diagnostic Pathology, Yahaba-cho, Shiwa-gun, Iwate, Japan; Departments of Molecular Diagnostic Pathology, Yahaba-cho, Shiwa-gun, Iwate, Japan; Departments of Molecular Diagnostic Pathology, Yahaba-cho, Shiwa-gun, Iwate, Japan; Departments of Molecular Diagnostic Pathology, Yahaba-cho, Shiwa-gun, Iwate, Japan; Head and Neck Surgert, Iwate Medical University, Yahaba-cho, Shiwa-gun, Iwate, Japan; Departments of Molecular Diagnostic Pathology, Yahaba-cho, Shiwa-gun, Iwate, Japan

## Abstract

SWItch/Sucrose Non-Fermentable (SWI/SNF) -related matrix-associated actin-dependent regulator of chromatin (SMARC) subfamily B member 1 (SMARCB1) deficient sinonasal carcinoma (SdSNC) is a rare variant of sinonasal undifferentiated carcinoma (SNUC). A 72-year-old man was referred to our hospital with complaints of left facial pain and nasal obstruction. Computed tomography (CI) revealed a tumor 5.5 cm in size in the left nasal cavity. Atypical cells with eosinophilic cytoplasm proliferating as solid nests and exhibiting necrosis were observed and diagnosed as poorly differentiated carcinoma. Carbon ion radiotherapy was performed. Follow-up CI revealed multiple masses in both lungs. Partial resection of the right lung was performed. Proliferating atypical cells with clear-to-eosinophilic cytoplasm were observed and resembled those in the paranasal sinus tumor. Immunohistochemical analysis indicated a metastatic lung tumor derived from the SNUC revealed completely negative SMARCB1 expression in the nuclei of the tumor cells. SdSNC is difficult to diagnose. However, molecular targeted therapy may be useful. Thus, it is necessary and important to recognize this rare cancer accurately.

## INTRODUCTION

Sinonasal undifferentiated carcinoma (SNUC) is defined as an undifferentiated carcinoma of the sinonasal tract without glandular or squamous features that is not otherwise classifiable [1]. SNUC is rare, with an incidence of ~0.02 per 100 000 people, and accounts for only ~3–5% of all sinonasal carcinomas [2]. Recent advances in molecular and immunohistochemical techniques have identified distinct entities that were previously classified as SNUC. These entities include sinonasal carcinoma deficient in SWItch/Sucrose Non-Fermentable (SWI/SNF) related matrix-associated actin-dependent regulator of chromatin (SMARC) subfamily B member 1 (SMARCB1; also known as SNF5 and INI-1) [3].

## CASE REPORT

**Figure 1:**
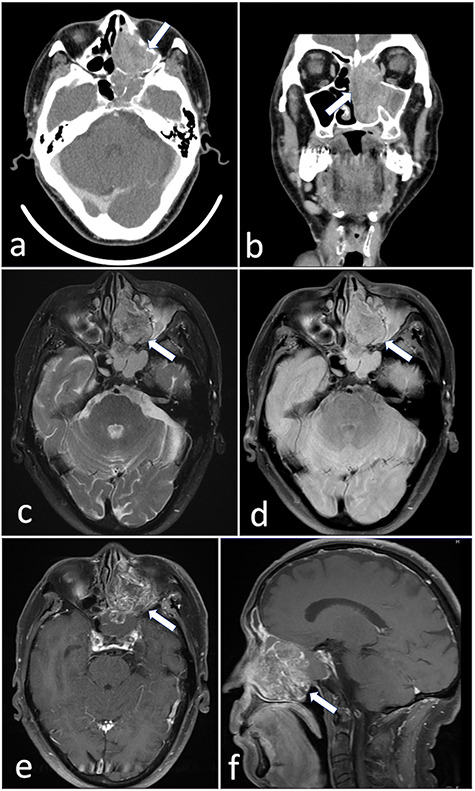
(**a** and **b**) CT revealed a tumor 5.5 cm in size in the left nasal cavity (arrow). (**c**–**f**) Magnetic resonance imaging revealed a tumor 5.5 × 4 × 3 cm in size occupying the left ethmoidal sinus and left nasal cavity, with invasion of the left orbital cavity, left maxillary sinus and intracranial cavity (arrow).

**Figure 2:**
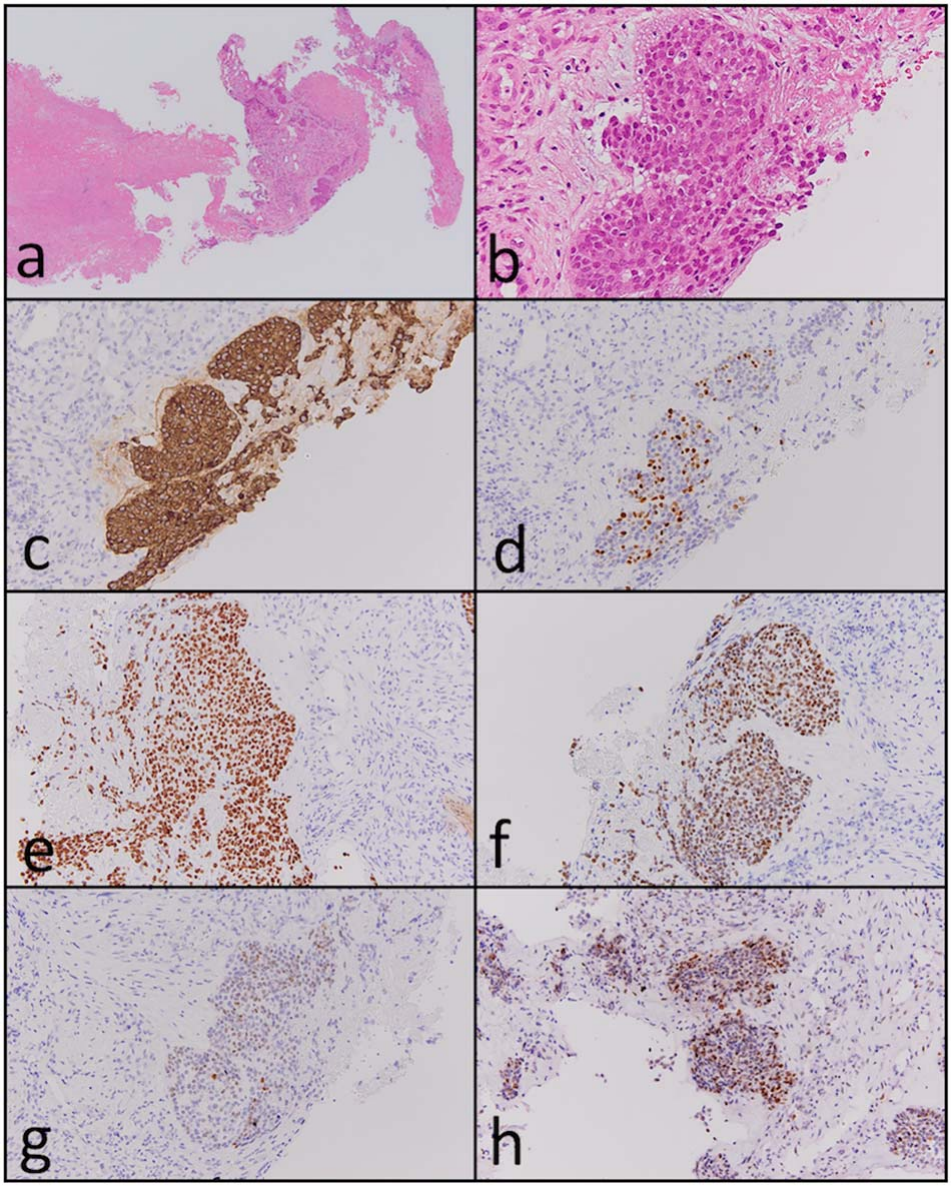
Histopathological and immunohistochemical findings of the paranasal sinus tumor. (**a** and **b**) Atypical cells with eosinophilic cytoplasm proliferating in a solid nest pattern and displaying massive necrosis were observed (hematoxylin and eosin staining, ×40 and ×400). The tumor cells were positive for (**c**) cytokeratin AE1/AE3 (×200), (**d**) p63 (×200), (**e**) Sox-2 (×200), (**f**) SALL4 (×200), (**g**) CDX-2 (×200) and (**h**) p53 (×200).

A 72-year-old man was referred to our hospital with complaints of left facial pain and nasal obstruction. Upon physical examination, a mass lesion was found in the left nasal cavity. Computed tomography (CT) revealed a tumor 5.5 cm in size in the left nasal cavity (Fig. 1a and b). Magnetic resonance imaging revealed a tumor 5.5 × 4 × 3 cm in size in the left ethmoidal sinus and left nasal cavity, with invasion of the left orbital cavity, left maxillary sinus and intracranial cavity (Fig. 1c–f). A tumor biopsy was performed. Histopathologically, atypical cells with eosinophilic cytoplasm proliferating as solid nests were apparent (Fig. 2a and b). Massive necrosis was also seen. Immunohistochemistry was performed using the Dako Envision+ system with dextran polymers conjugated to horseradish peroxidase (Dako, Glostrup, Denmark). The following primary antibodies were used: CDX-2 (DAK-CDX2, Dako), chromogranin A (polyclonal, Abcam, Cambridge, England), cytokeratin (AE1/AE3, Dako), desmin (D33, Dako), NCAM (123C3, Dako), NUT (polyclonal, Abcam), p16 (E6H4, Roche, Basel, Switzerland), p53 (DO7, Dako), p63 (DAK-p63, Dako), SALL4 (6E3, Sigma-Aldrich, St Louis, MO, USA), SMARCA4 (polyclonal, Proteintech, Rosemont, IL, USA), SMARCB1 (polyclonal, Bethyl Laboratories, Montgomery, TX, USA), Sox-2 (D1C7J, Cell Signaling Technology, Danvers, MA, USA), synaptophysin (DAK-SYNAP, Dako) and S-100 (polyclonal, Dako). The tumor cells were positive for cytokeratin AE1/AE3 (Fig. 2c) and p63 (Fig. 2d) but negative for S-100, NCAM, synaptophysin and chromogranin A. The tumor was diagnosed as poorly differentiated carcinoma of the paranasal sinuses at first time.

**Figure 3:**
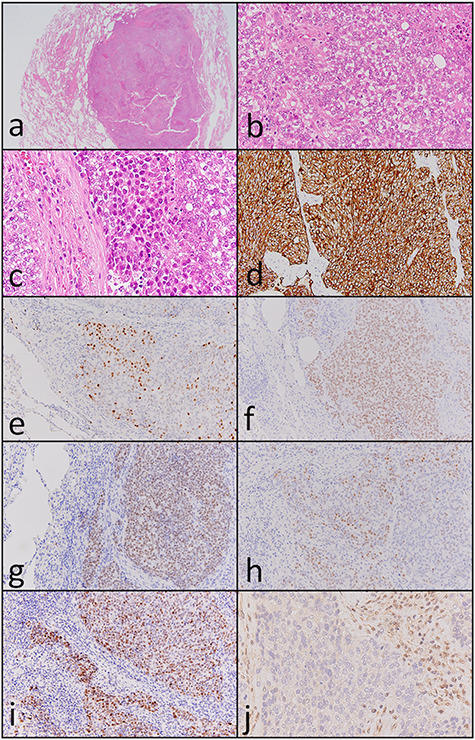
Histopathological and immunohistochemical findings of the lung tumor. (**a** and **b**) Atypical cells with clear-to-slightly eosinophilic cytoplasm proliferating in a solid nest pattern and displaying comedo necrosis (hematoxylin and eosin staining, ×40 and ×400). (**c**) Some of the atypical cells showed a ‘rhabdoid’ morphology such as eccentric nuclei (hematoxylin and eosin staining, ×400). Tumor cells were positive for (**d**) cytokeratin AE1/AE3 (×200), (**e**) p63 (×200), (**f**) Sox-2 (×200), (**g**) SALL4 (×200), (**h**) CDX-2 (×200) and (**i**) p53 (×200). (**j**) SMARCB1 expression was completely absent in the nuclei of the tumor cells but was observed in the nuclei of surrounding non-neoplastic cells (×400).

Carbon ion radiotherapy was performed at another hospital. Subsequently, follow-up CT revealed multiple masses in both lungs. Partial resection of the right lung was performed. Histopathologically, atypical cells with clear-to-slightly eosinophilic cytoplasm proliferating as solid nests and exhibiting comedo necrosis were observed (Fig. 3a and b). Some of the atypical cells had a ‘rhabdoid’ morphology such as eccentric nuclei (Fig. 3c); they resembled the atypical cells of the paranasal sinus tumor histopathologically. Immunohistochemically, tumor cells in the resected lung specimen were positive for cytokeratin AE1/AE3 (Fig. 3d), p63 (Fig. 3e), Sox-2 (Fig. 3f), SALL4 (Fig. 3g), CDX-2 (Fig. 3h) and p53 (Fig. 3i), and were negative for p16, NCAM, synaptophysin, chromogranin A and S-100. In addition, the paranasal sinus tumor was positive for Sox-2 (Fig. 2e), SALL4 (Fig. 2f), CDX-2 (Fig. 2g) and p53 (Fig. 2h). Thus, these tumors were diagnosed as SNUC of the paranasal sinuses and lung metastasis originating from the SNUC. Subsequently, immunohistochemical analyses of SMARCB1, SMARCA4 and NUT were performed and showed that SMARCB1 was completely absent in the nuclei of the tumor cells but was expressed in the nuclei of the surrounding non-neoplastic cells (Fig. 3j). Both SMARCA4 and NUT expression was normal. Although the size of the paranasal sinus tumor immediately showed complete response, brain metastasis subsequently developed and the patient ultimately died.

## DISCUSSION

SMARCB1-deficient sinonasal carcinoma (SdSNC), which was first reported in 2014 by Agaimy *et al.* and Bishop *et al.* independently, is characterized by SMARCB1 protein loss and somatic SMARCB1 gene deletion and shows very aggressive behavior [4, 5]. Since then, several reports on SdSNC have been published, including a recent systematic literature review published by Parsel *et al.* [6]. According to that review, the age at SdSNC presentation ranges from 19 to 89 years, and there is a slight male predilection; the most common diagnosis is SNUC, and surgical resection with adjuvant radiation or chemoradiation therapy is the treatment of choice [6]. Overall, the prognosis is poor, as most tumors are diagnosed at an advanced stage, and the average mortality rate is 45.3% [6]. That review reported a median (range) overall survival of 22 (12–44) months [6]. Two studies compared SdSNC with other sinonasal malignancies [6]. According to those comparisons, the rate of recurrence was 17–53% for SNUC with preserved SMARCB1 expression compared with 53–60% for SdSNC [6]. Thus, it is very important to differentiate SdSNC from SNUC because the treatment selections differ. Two specific EZH2 inhibitors, EI1 and EPZ-6438, have shown promise in treating SMARCB1-deficient tumors [7]. Phase II clinical trials of an oral EZH2 inhibitor are currently underway for both adults and pediatric patients with SMARCB1-negative tumors (https://www.cancer.gov/about-cancer/treatment/clinical-trials/intervention/tazemetostat) [8]. Correctly identifying all forms of SdSNC will potentially allow patients to benefit from such targeted therapies.

Histopathologically, Agaimy *et al.* reported that most tumors display either a predominantly basaloid (61%) or plasmacytoid/rhabdoid (36%) morphology [3]. The plasmacytoid/rhabdoid variant consists of sheets of tumor cells with abundant, eccentrically placed eosinophilic cytoplasm, whereas such cells are typically rare and singly distributed in the basaloid variant. Since that report, Ayyanar *et al.* argued that as the number of reported cases has increased, the morphological spectrum of this tumor has gradually expanded [9]. Many different morphological features have been described in the recent literature. Agaimy *et al.* described squamoid, spindle cell, sarcomatoid and adenoid variants [3], and Wasserman *et al.* described focal clear cell features and the presence of non-specific empty vacuoles [10]. In our case, we could not detect any specific appearance, such as basaloid and/or rhabdoid/plasmacytoid morphology, in the primary sinonasal lesion because of the small biopsy specimen. On the other hand, clear cell morphology was found in most of the metastatic lesion, and a plasmacytoid/rhabdoid morphology was slightly found in our case.

In conclusion, SdSNC is rare with a poor prognosis. In addition, the diagnosis is difficult. However, molecular targeted therapy may be useful. Thus, it is necessary and important to recognize this rare cancer accurately.
